# A novel bioassay for quantification of surface Cannabinoid receptor 1 expression

**DOI:** 10.1038/s41598-020-75331-y

**Published:** 2020-10-23

**Authors:** Ismael Rodríguez-Rodríguez, Joanna Kalafut, Arkadiusz Czerwonka, Adolfo Rivero-Müller

**Affiliations:** 1 Department of Organic Chemistry, Faculty of Chemistry, Jagiellonian University, Krakow, Poland; 2 Department of Biochemistry and Molecular Biology, Medical University of Lublin, Lublin, Poland; 3 Department of Virology and Immunology, Faculty of Biology and Biotechnology, Maria Curie-Skłodowska University, Lublin, Poland

**Keywords:** Gene expression analysis, Molecular engineering, Sensors and probes, Biological techniques, Cell biology, Drug discovery, Molecular biology, Structural biology, Medical research, Molecular medicine

## Abstract

The cannabinoid receptor type 1 (CB1) plays critical roles in multiple physiological processes such as pain perception, brain development and body temperature regulation. Mutations on this gene (*CNR1*), results in altered functionality and/or biosynthesis such as reduced membrane expression, changes in mRNA stability or changes in downstream signaling that act as triggers for diseases such as obesity, Parkinson’s, Huntington’s, among others; thus, it is considered as a potential pharmacological target. To date, multiple quantification methods have been employed to determine how these mutations affect receptor expression and localization; however, they present serious disadvantages that may arise quantifying errors. Here, we describe a sensitive bioassay to quantify receptor surface expression; in this bioassay the *Gaussia* Luciferase (GLuc) was fused to the extracellular portion of the CB1. The GLuc activity was assessed by coelenterazine addition to the medium followed by immediate readout. Based on GLuc activity assay, we show that the GLuc signals corelate with CB1 localization, besides, we showed the assay’s functionality and reliability by comparing its results with those generated by previously reported mutations on the *CNR1* gene and by using flow cytometry to determine the cell surface receptor expression. Detection of membrane-bound CB1, and potentially other GPCRs, is able to quickly screen for receptor levels and help to understand the effect of clinically relevant mutations or polymorphisms.

## Introduction

The cannabinoid receptor 1 (CB1), a member of the G protein-coupled receptor (GPCR) family, is an important member of the endocannabinoid system and is known to be expressed in the Central Nervous System (CNS)^1^ and multiple other tissues and cell types^2^. In the CNS, the CB1 plays a key role in multiple processes such as pain perception, brain development and body temperature regulation^1,2^. It has been widely studied and is considered a potential pharmacological target for multiple diseases, including neuropsychological and neurodegenerative disorders^2^.

This receptor is encoded by the *CNR1* gene, which in humans is located at the chromosome 6 (6q14-q15), this gene is comprised by 4 exons; although the entire coding region is contained within exon 4^3^. CB1 expression levels are known to variate during embryo neurodevelopment^4^ and other development stages^3^. Altered expression of this receptor is directly linked to some pathological conditions such as Huntington’s^5^, Alzheimer’s^6^, Parkinson’s diseases^7^, obesity and diabetes^8^. Multiple mechanisms have been reported to be responsible for the expression of CB1, causing a broad number of dysregulation effects depending on the affected tissue^9^. The regulation of the expression of this receptor represents one of the major therapeutic target for the development of new drugs that could restore its normal physiological levels^3,10^. Modulation of CB1 expression has also been observed upon prolonged endocannabinoids exposure, such as the case of Neuropathic pain, where the CB1 expression is downregulated^11^, as well as under pharmacological modulation^12^.

Variants of *CNB1* have been found to result in changes in the amount of the expressed CB1, such as the rs1406977 SNP, carriers of this allele (G) have reduced *CNR1* prefrontal mRNA expression amount compared with A/A subjects^13^. Other polymorphisms might not alter receptor expression but its affinity and response to agonists, that is the case of the rs2023239 SNP, a potential biomarker for susceptibility to cannabis use, which causes an alternative splicing of the *CNR1* increasing CB1 affinity for its agonists^14^. To personalize treatments, there is need to analyze changes of expression caused by different polymorphisms or mutations. An example of the change in CB1 localization is point mutation F237L that reduces the membrane expression of this receptor^15^. Mutation T210I is characterized not only by reduced membrane expression but also by several times greater affinity for ligands, making it essentially constitutively active^16^, while mutation T210A results in a completely inactive CB1 despite membrane localization, although it seems to lack internalization capabilities^17^.

Many techniques have been developed and employed for the quantification of GPCRs cell surface expression; the most common include flow cytometry using fluorescent probes^18,19^, ELISA^20^, surface biotinylation^21^, among others, which require long incubations and multiple washing steps that increase quantitative determination variability per assay, or involve complex imaging equipment. In order to solve these problems, here we report an inexpensive and easy bioassay for the relative quantification of the CB1 receptor expression at the cell surface; it is based on the genetic fusion of the CB1 and *Gaussia* luciferase (GLuc), a widely used enzymatic reporter in eukaryotes. As a proof of principle, we analyzed three previously reported point mutations (CB1^F237L^, CB1^T210I^ and CB1^T210A^) and compared them to the wild-type (WT) counterpart^22^.

## Materials and methods

### Materials

KOD-Xtreme hot-start DNA polymerase (Merck Millipore), tiHybrid and Hybrid DNA Polymerases (EURx), SsoFast EvaGreen Supermix (BIO RAD), DreamTaq Green PCR Master Mix and Turbofect Transfection Reagent (ThermoFisher Scientific), Restriction endonuclease *DpnI*, *EcoRI*, and Gibson Assembly Master Mix (NEB), the plasmid used for substitution (GLuc-TEV-CBD) was designed in our laboratory, pmR-Cherry (Clontech), Coelenterazine (Selleckchem), human embryonic kidney-293T (HEK293T) cells (ATCC), Dulbecco’s Modified Eagle Medium (DMEM)/F12 medium (Gibco), fetal bovine serum (FBS; PromoCell), ampicillin (Polfa Tarchomin), penicillin and streptomycin (Sigma Aldrich), DYKDDDDK Tag (9A3) Mouse mAb (Cell Signaling), Donkey Anti-Mouse IgG H&L Alexa Fluor 555 (Invitrogen).

### Plasmid design

A plasmid containing the *CNR1* gene fused to the *GLuc* gene (*GLuc-CNR1*) was generated by Gibson Assembly by combining products from two PCR reactions previously carried out to amplify the *CNR1* gene (F_1_: AAGTCGATCCTAGATGGCCTTG, R_1_: TCTTAAGGAGGGATGGGGTGA F_2_: AAAAATACTGACTCCAACCATTCAA R_2_: TTTGCCATCAGACTGTGAAATAAGG). The *CRN1* gene was cloned from genomic DNA isolated from a mouth-swab of the main author (IR-R), using the previously mentioned primers to amplify the coding region and the long 3′-UTR (5.4 Kb) fragments. Linearized vector was digested with *DpnI* to remove methylated DNA and purified using purification columns (DNA Clean and Concentrator Kits, ZYMO RESEARCH). The generated plasmid sequence was designed to provide both proteins (CB1 and GLuc) with a high rotational flexibility and enabling immunodetection by adding the sequence FLAG tag between them. The CRN1-FLAG-GLuc plasmid was then used for generating the *CNR1* mutants (CB1^F237L^, CB1^T210I^ and CB1^T210A^).

### Construct description

**Figure 1:**
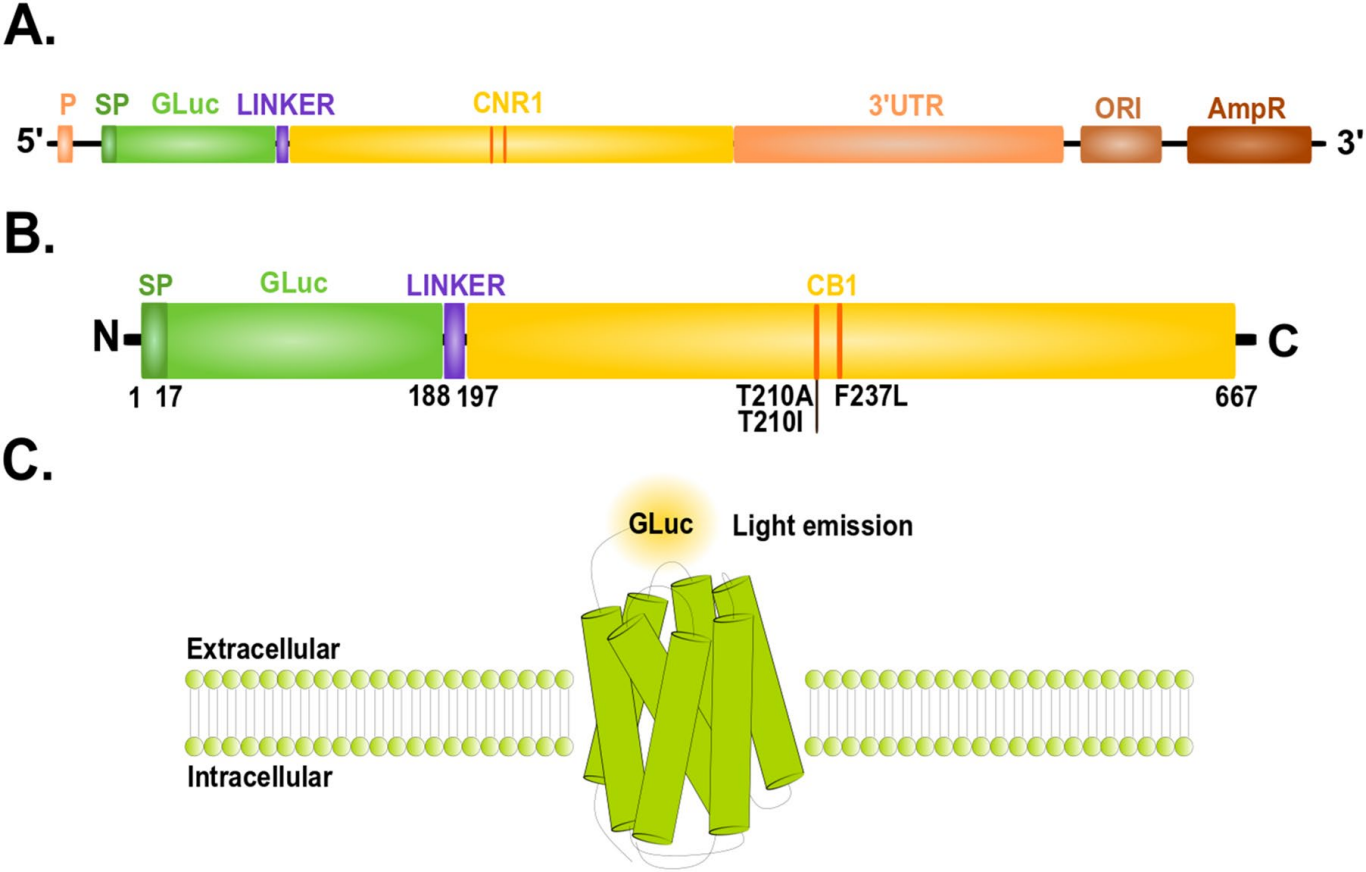
Schematic overview of the engineered system and resulting protein used throughout this study. (**A**) The vector is designed to express *GLuc* with full-length *CNR1* linked by a short sequence (linker) which was either the flexible domain (GLEG) or a FLAG tag (DYKDDDDK). The vector also contains the endogenous long 3′UTR of *CNR1*; (**B**) resulting protein containing GLuc (with signaling peptide, SP) fused via a linker to the N terminus of CB1 receptor. *P* promoter, *SP* signaling peptide, *CNR1*- cannabinoid receptor 1 gene, *CB1-* cannabinoid receptor 1 protein, Linker: either flexible domain (GLEG) or *FLAG* tag sequence (DYKDDDDK), *3′UTR *untranslated region, *ORI* origin of replication, *AmpR* ampicillin resistance. (**C**) Schematic representation of the proposed CB1 quantification bioassay. CB1 fused to GLuc is measured by luciferase activity under the presence of coelenterazine.

The architecture of the constructs consisted of the *GLuc* gene, which is comprised of 558 bp, cloned directly at the N-terminal of the *CNR1* gene. To ensure proper folding and functioning they were linked by either a flexible domain (GLEG) or the FLAG sequence (DYKDDDDK), the latter for immunodetection purposes. The full-length *CNR1* (consisting of 1416 bp) followed by the naïve long 3′-UTR of the coding sequence (3992 bp) was as well cloned into the vector. The 3′UTR of *CNR1* has been shown to influence the expression levels of this receptor^23,24^. The signal peptide used to localize both proteins at the membrane is contained within the *GLuc* coding sequence (1 –17 amino acids). Schematic plasmid vector and protein construct is shown on Fig. 1.

### Mutagenesis

The *GLuc-FLAG-CNR1* plasmid was used for generating the *CNR1* mutants (CB1^F237L^, CB1^T210I^ and CB1^T210A^). Based on this sequence, a pair of inverse PCR primers for mutagenesis were designed. The sequences of the forward and reverse primers for CB1^F237L^ are 5′-GCCGTGGTGGCGTTATGCCTGATGTGGACCATAG-3′ and 5′-AGGCATAACGCCACCACGGCCTTGG-3′, for CB1^T210I^ are 5′-GTTCCTCATAGCCATCGACAGGTACATATC-3′ and 5′-CCTGTCGATGGCTATGAGGAACAGGCTGCCCAC-3′ and for CB1^T210A^ are 5′-GTTCCTCGCAGCCATCGACAGGTAC-3′ and 5′-CTGTCGATGGCTGCGAGGAACAGGCTGCCCAC-3′, respectively.

### Transformation and plasmid purification

Previously prepared electrocompetent *E. coli* bacteria^25^ were electroporated with the plasmid generated by Gibson Assembly; resulting colonies (after selection with ampicillin 100 mg/mL) were checked by colony PCR. Positive colonies were then digested with *EcoRI*, those showing the expected length (10,146 bp) were sequenced. At least two sequence-verified clones for each construct were used for the experiments.

Plasmid DNA (pDNA) was isolated by minipreps (Zyppy Plasmid Miniprep Kit, ZYMO RESEARCH). Isolated plasmid’s integrity was observed in 1% Agarose gel; enough plasmid concentration was isolated and stored at − 20 °C for until further use. The *GLuc-linker-CNR1* plasmids purity and concentration were determined by UV spectroscopy, a concentration of 540.2 ng/µL was obtained with a A_260_/A_280_ ratio of 1.8 indicating a high purity. Plasmids carrying mutations of *CNR1* showed similar concentrations and A_260_/A_280_ ratios.

### Transfection and receptor quantification

HEK293T were cultured in DMEM/F12 culture medium supplemented with 10% FBS and 1% antibiotics at 37 °C and 5% CO_2_ saturation. The cells were then seeded in a 96-well plate (1 × 10^4^ cells/well), 24-well plate (5 × 10^4^ cells/well) or 6-well plates (2 × 10^5^ cells/well) and transfected with the *CNR1*-GLuc plasmid using Turbofect Transfection Reagent following the manufacturer’s protocol. The DNA amounts used for the transfections for both; luciferase assay and flow cytometry were in accordance with those indicated by the manufacturer’s protocol and are as follows: 0,2 μg per well in 96-well plates, 1 μg per well in 24-well plates and 4 μg in 6-well plates. Transfections were performed in triplicate.

### Receptor relative quantification

To quantify the expression of the CB1 receptor, the GLuc detection assay was performed 48 h after transfection. The culture medium was transferred to another well and each well containing transfected HEK293T cells was washed multiple times with PBS. A solution of 20 µM coelenterazine was diluted in 5 mL PBS and incubated for 30 min at room temperature under dark to allow its stabilization. Subsequently, the final 10 µM concentration of cell-added coelenterazine was obtained by mixing 50 µL of the stable coelenterazine with 50 µL of the cells medium. The GLuc activity was measured immediately by triplicate using a plate luminometer (TECAN Infinite 200 PRO); results were then statistically analyzed.

### Flow cytometry analyses

GLuc-CB1 detection in the cells was assessed by measurement of the fluorescence intensity from binding the primary anti-FLAG mouse antibody (Cell Signaling Technology, Cat. Nr: #8146) and secondary anti-mouse antibody conjugated with Alexa Fluor 555 dye (Invitrogen Cat. Nr: A-31570). The HEK293T cells were seeded onto 6-well plates 24 h before transfection. Transfection procedure was described at *Transfection and receptor quantification* section. After 48 h, the cells were washed (Ca^2+^ and Mg^2+^-free PBS) and harvested (5 mM solution of EDTA in Ca^2+^ and Mg^2+^-free PBS). Both, free floating as well as adherent cells were gathered. Next, after centrifugation (500×*g*, 5 min), the appropriate cells pellets were separated into two main parts.

The part of cells was used for total (intracellular and surface membrane) CB1 detection. Cells were fixed by resuspending in fixation and permeabilization buffer (BD Pharmingen, Cytofix/Cytoperm solution, cat. numb. 554722) and incubated for 20 min on ice. Next, cells were washed (BD Perm/Wash buffer, cat. numb. 554723) and centrifuged (500×*g*, 5 min). Mocked (UT), CB1^F237L^, CB1^T210I^ and CB1^T210A^ cells were incubated with primary anti-flag antibody (1 h, 37 °C and 5% CO_2_) and subsequently after wash step, labeled with secondary Alexa Fluor 555-conjugated antibody (1 h, 37 °C and 5% CO_2_). Part of the cells were incubated only with Alexa Fluor 555 conjugated Ig (described as control). For total CB1 quantification all Ig dilutions and wash steps were made in BD Perm/Wash buffer.

The second part of harvested cells were used for surface membrane CB1 detection. The cell fixation and permeabilization steps were omitted. Next, cells were prepared as described above. All Ig dilutions and wash steps for surface membrane CB1 detection were made in Ca^2+^ and Mg^2+^-free PBS.

All stainings were performed directly before the flow cytometric analysis (BD FACSCalibur, CellQuest Pro Version 6.0. software for the Macintosh operating system). The fluorescence Alexa Fluor 555 dye intensity of individual cell was determined and at least 10,000 events were measured within an acquisition rate of 100–300 events/s.

### Statistical analyses

Significance among luminescence readings was assessed by one-way ANOVA followed by Tukey’s *post-hoc* test. Statistical analyses of the flow cytometry were performed using GraphPad Prism 8.0 (GraphPad Software Inc., California, U.S.A). ANOVA with Tukey post hoc test and column statistics were used for comparisons (*, p < 0.05; **, p < 0.01; ***, p < 0.001 was considered statistically significant). All tests were performed in the triplicates, at least.

## Results and discussion

We took advantage of the natural properties of GLuc which does not require cofactors and it is normally secreted—it contains a secretion signal peptide^26^. This allows us to quantify the CB1 relative expression on the surface of HEK293T cells. The relative quantification is easily done by measuring the luminescence generated by the GLuc enzyme linked to the CB1 protein, since this luminescence depends on the amount of receptor, changes on its expression will be quantifiably noted. A schematic representation of this method is shown in Fig. 1C.

As described in the *Construct Description* section, the signal peptide (SP in Fig. 1) used to localize the receptor at the membrane is encoded in the *GLuc* gene, in agreement with previous research works that have employed artificial signal sequences at the N-terminus to allow membrane localization^27^ since the majority of the GPCR receptors including CB1 do not possess a signal peptide to be trafficked to the cell surface^28^. Foreign sequences at the N-terminal of GPCRs, are known to affect the addressing of the receptor to the cell surface due to a low efficiency of translocation through the endoplasmic reticulum (ER). For this reason, the addition of a signal sequence (signal peptide, SP) at the N-terminus of a fusion construct is necessary^27,29^. In this case, GLuc, which already contains a SP, was inserted in front of CB1 separated by a short linker to avoid interfering with the CB1 folding and topology; in some other cases the fusion of such chimeric proteins has been done between residues 25 and 26 of the extracellular N terminus following the same principle^27^.

**Figure 2:**
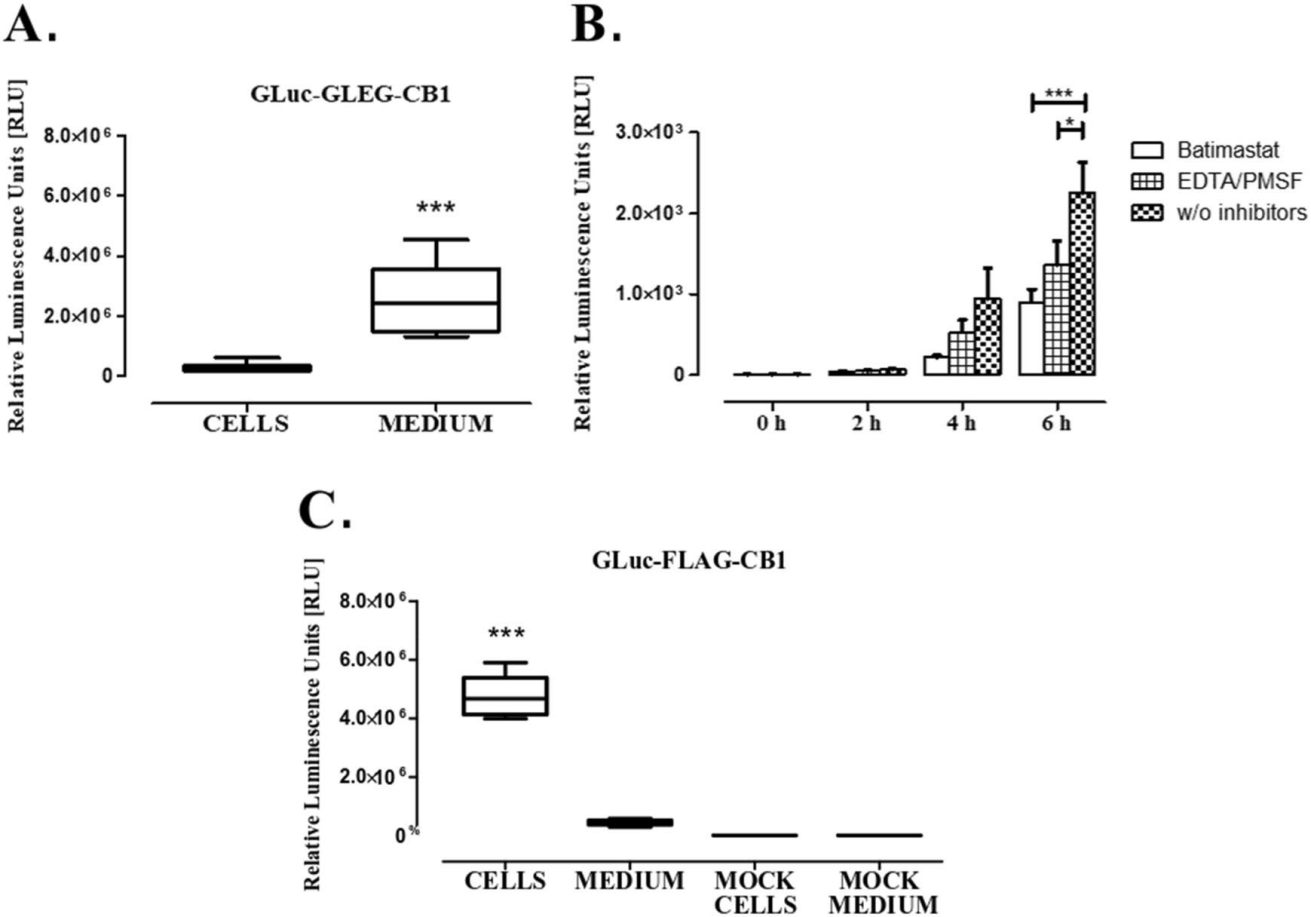
The construct GLuc-GLEG-CB1 contains a cleavage site that causes GLuc to be released into the medium. (**A**) Cells expressing the GLuc-GLEG-CB1 were tested 48 h after transfection by adding coelenterazine, it can be observed how the luminescence is originating from the medium and not from the cells, suggesting that the generated GLuc is being cleaved and released into the medium. (**B**) The RLU level in culture medium after protease inhibitors treatment of transfected cells. The incubation of cells expressing GLuc-GLEG-CB1 with different protease inhibitors at fixed concentrations (Batimastat 1 µM, EDTA 2 mM, PMSF 0.2 mM) and time points (0 h, 2 h, 4 h and 6 h) suggests that the linker sequence contains a metalloprotease cleavage site (metalloprotease predicted site is presented in Supplementary Fig. S1), as it can be observed from the readings coming from the medium of transfected cells. (**C**) In contrast, 48 h after transfection luminescence from cells expressing the construct GLuc-FLAG-CB1 comes entirely from the cells, while virtually no signal was obtained from their medium. The RLU level for mock transfected cells (mock cells) and their medium (mock medium) is shown in the Fig. 2C. The results represent the mean ± SD analyzed with one-way ANOVA test and Tukey’s multiple comparison post-hoc test vs. the mock cells and medium control (**A**,**C**).

At first, the initial architecture containing the GLuc linked to the CB1 by a flexible domain consisting of 4 amino acids (GLEG), resulted in that most of GLuc was found in the medium and not attached to the cells (Fig. 2A), what seems to be caused by the cleavage of the GLuc-CB1 by a protease. To address this, we incubated cells expressing this GLuc-CB1 with several protease inhibitors and found the Batimastat, a pan-metalloprotease inhibitor, diminished the amount of free GLuc (Fig. 2B). We then analyzed a short sequence that comprises the GLEG flexible domain and the proteins GLuc and CB1 (specifically DKIKGAGGDGLEGKSILD) for metalloprotease sites using a prediction software “SitePrediction” (https://www.dmbr.ugent.be/prx/bioit2-public/SitePrediction/) and found a potential cleavage site that could disrupt the generated chimeric protein causing GLuc enzyme to be released (Supplementary Fig. S1). This sequence was partially removed by inserting a FLAG Tag between the GLuc and the CB1, which plays the role of a linker and a spacer for proper folding Fig. 1. Subsequent experiments showed that the GLuc activity, after cells were transfected with the *GLuc-FLAG-CNR1* plasmid, was virtually only from cells (Cells) and not in the culture medium (Medium) (Fig. 2C). Mock transfected cells and their medium showed baseline luminescence values, as shown in Fig. 2C. Based on the results, the generated luminescence specifically comes from the transfected cells, clearly demonstrating that the enzyme is not being secreted and that it is only readable in cells expressing the GLuc-FLAG-CB1. Full sequences of both architectures (*GLuc-FLAG-CNR1* and *GLuc-GLEG-CNR1*) are provided in the Supplementary material, both as nucleic acids and amino acid sequences, as well, the plasmid maps are shown in Supplementary Figs. S2 and S3.

The above results show that adding a FLAG tag between the GLuc and the CB1 let the chimeric protein be expressed at the cell surface and being properly folded. This also suggests that any intracellular GLuc signal is minimal e.g. during biosynthesis and localization, as proteolytically processed GLuc would show activity within the cells while transporting, which is virtually unperceived.

The one-way ANOVA showed that there is a statistically significant difference among the means of the four factors (cells and medium from transfected GLuc-FLAG-CB1 and mock transfected cells) (p ≤ 0.001); the Tukey’s test revealed that the transfected cells (Cells) mean is significantly different from those of the other three factors, confirming the source of luminescence.

**Figure 3:**
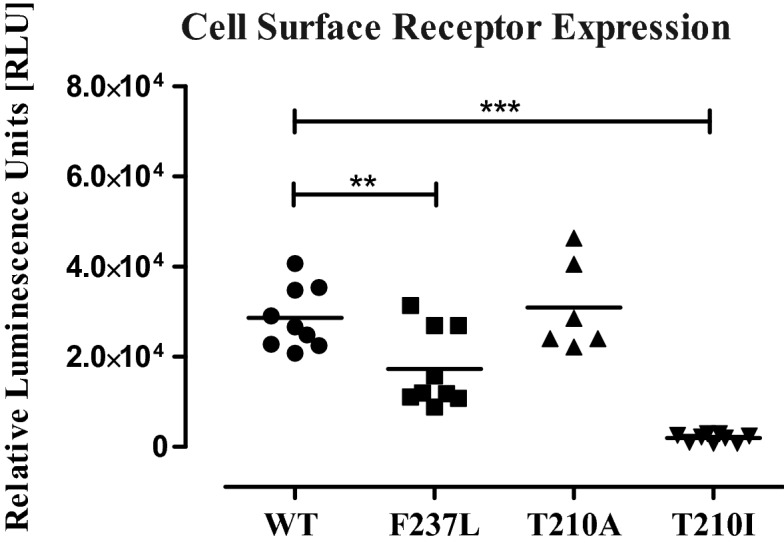
WT and mutant CB1 relative expression on the surface of HEK293T cells. Cells expressing GLuc-FLAG-CB1^WT^ (WT), GLuc-FLAG-CB1^F237L^ (F237L), GLuc-FLAG-CB1^T210I^ (T210A) and GLuc-FLAG-CB1^T210A^ (T210I) are shown, in order to generate a read-out, coelenterazine was added to the cells after medium was washed away. Similar expression levels can be observed for CB1^T210A^ and the CB1^WT^, showing that this mutation does not affect the expression of the receptor, however, mutations CB1^F237L^ and CB1^T210I^ reduced the expression at the surface of HEK293T cells, as compared to the CB1^WT^.

Having established that the receptor is at the cell membrane and can be quantified through this bioassay, we next generated a series of *CNR1* mutants (CB1^F237L^, CB1^T210I^, CB1^T210A^), in order to demonstrate changes in the cell surface expression caused by these point mutations are in line with previous observations. Indeed, the mutants behaved as expected, where the CB1^T210A^ exhibited similar expression to CB1^WT^, while CB1^F237L^ and CB1^T210I^ showed considerable reduced expression on membrane (Fig. 3).

The GLuc-CB1 reporter showed that there is a statistically significant difference among the means of the cells transfected by GLuc-FLAG-CB1^WT^ and GLuc-FLAG-CB1^F237L^, or GLuc-FLAG-CB1^T210I^ (Fig. 3), however, no difference was observed between those transfected by GLuc-FLAG-CB1^WT^ and GLuc-FLAG-CB1^T210A^, indicating similar cell surface receptor expression. Figures 2C and 3 also show the importance of running all controls [positive (WT) and negative] within the same run, as changes in cell number, cells state, reagents batch and other environmental factors might significantly affect the readout, as can be observed by a reduction in the RLU units obtained in the two different experiments (4 × 10^6^ in Fig. 2C vs 3 × 10^4^ in Fig. 3). These differences should not be considered as a 100-fold reduction in the expression but are in fact inter assay variations. Comparisons can only be done between samples of the same run, as we have done for WT vs mutants in Fig. 3.

Through this bioassay we corroborated previous data in that the CB1^F237L^ and CB1^T210I^ mutants cause a reduced CB1 receptor expression level on the cell surface^15,16^. Our data also supports that CB1^T210A^ mutation results in unaffected expression^17^.

**Figure 4:**
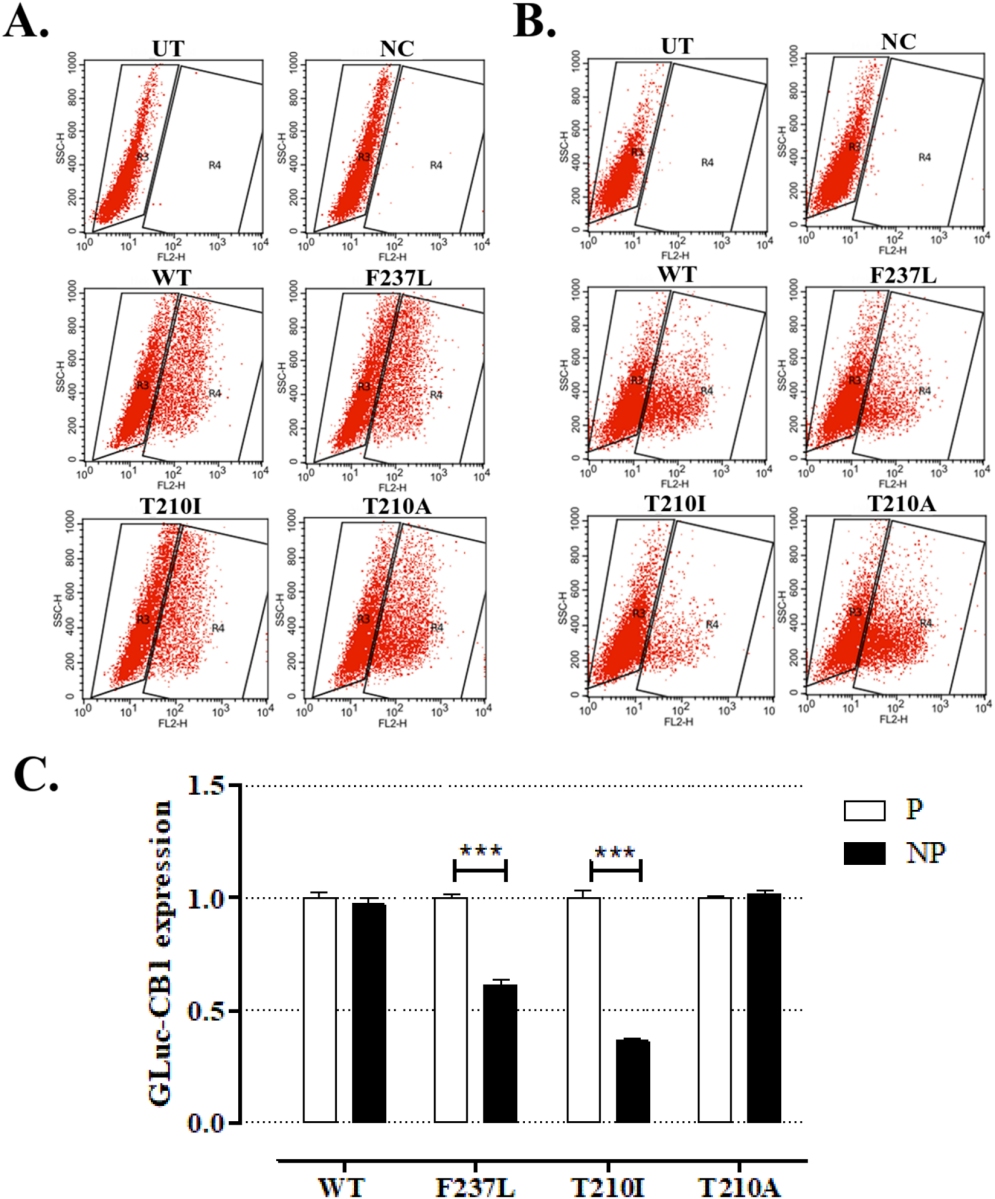
The total and surface-only level of GLuc-CB1 in HEK293T cells. The representative SSC-H/FL2-H 2D-dot plots for (**A**) permeabilized and (**B**) non-permeabilized wild type (WT), GLuc-FLAG-CB1^F237L^ (F237L), GLuc-FLAG-CB1^T210I^ (T210I) and GLuc-FLAG-CB1^T210A^ (T210A) cells are shown. The cells treated with secondary antibody only (NC) and not transfected by plasmids (UT) were used as negative controls for gating. (**C**) The median expression [the mean ± SD (n = 3)] of HEK293T cells classified as GLuc-CB1-positive (gate R4) in permeabilized (P) and non-permeabilized (NP) HEK293T cells normalized vs 1 are shown.

Next, we tested whether luciferase readouts agreed with membrane-anchored GLuc-FLAG-CB1 expression using an antibody against the FLAG tag by flow cytometry. The analyses are shown in Fig. 4. The CB1^WT^ and CB1^T210A^ mutant showed similar GLuc-CB1 expression both in permeabilized as well as in not permeabilized cells, while CB1^F237L^ and CB1^T210I^ have significantly lower (p ≤ 0.001) membrane expression (Fig. 4C) as expected from the GLuc readouts. GLuc-CB1 expression in permeabilized (total expression) and not permeabilized (membrane-anchored only) cells is shown in Fig. 4A,B, respectively. See Supplementary Table S1 for a statistical analysis of the collected data.

According to the flow-cytometry results, the total expression for all the mutants was apparently the same as the wild type, however the results demonstrate how the point mutations CB1^F237L^ and CB1^T210I^ directly diminish the membrane-anchored receptor quantity. The membrane expression of the wild-type and CB1^T210A^ clearly indicate that the addition of the signal peptide aided the membrane localization of the receptor, and that the lower levels of expression seen for the mutants CB1^F237L^ and CB1^T210I^ are caused by different pathways, as alterations in biosynthesis, internalization, a phenomenon previously observed for the mutated receptor F237L^15^, or by a constitutive endocytosis. Either way, the bioassay is able to detect such changes which is essential for screening of hitherto mutations.

The results obtained by cell cytometry and GLuc-generated relative luminescence units were both in accordance with each other and those in literature, clearly indicating that the readouts generated by both experiments quantify correctly the receptor expressed at the cell membrane for all the analyzed cases. This is also supported by the GLuc-GLEG-CB1 test where virtually no intracellular readout was detected (Fig. 2A) and instead almost all luminescence is found in the culture media.

There are some limitations that should be considered; in this study we have used the CB1 to demonstrate that the luminescence comes only from those transfected cells; however, it has been reported that the expression of this receptor occurs at other intracellular structures such as the mitochondria^30^. In addition, coelenterazine has a lipophilic nature and its able cross the cell membrane; a non-permeable substrate is available^31^, although this is easily detected by flow cytometry or confocal microscopy if the mutant is suspected to be mostly intracellularly located. Another limitation is that our assay cannot assess CB1 activity, for which the system could be coupled to some of the others signaling assays such as TANGO^32^, GloSensor^33^, or other assays that measure cAMP, inositol phosphate or calcium accumulation methods^34^ and two measurements could be accounted for, expression vs activity.

## Conclusion

CB1 plays key homeostatic role in a variety of physiological processes, new tools have been constantly being designed to provide a better understanding of its mechanisms and variability. With our quantification assay, determining how mutations affect the receptor expression in a much easier way than commonly used methods will ease research regarding the endocannabinoid system and its alterations upon previously reported pathological conditions. Moreover, GLuc has been used for BRET and therefore it can be used to detect ligand–receptor or receptor-receptor interactions on the plasma membrane of living cells.

The main novelty of our work is determined by the fact that it could be possible to detect the effect of various alterations in the chemical environment (such as the constant binding of ligands) or genetical factors (such as the presence of point mutations or polymorphisms) on the GPCR receptors’ expression^14^.

The detection of membrane localization of particular GPCRs is fundamental to understand the roles of mutations and protein interactions. In fact, the altered expression of the CB1 receptor is in clear association with various mental-related issues such as major depression, schizophrenia, bipolar disorder, among others^35–37^. Our research could provide the foundation to better understanding how dysregulation of the membrane expression of CB1 plays a role in the molecular basis for the afore mentioned diseases.

Multiple mutations, polymorphisms, and alternative splice variants of the CB1 receptor have been identified and recognized as important pharmacological targets in multiple diseases, these genetic variants of the CB1 have been deeply described elsewhere^38^. Thus, screening how these genetic variants affect the level of expression of this and other GPCRs can speed up the diagnosis and treatment, with e.g. molecular chaperones^39^, of multiple genetic-based diseases.

As previously described, other quantifying methods including flow-cytometry or ELISA can be employed to determine surface expression of the CB1 and other GPCRs, however, through a simplified bioassay as the one described here, we offer a new simple methodology with results that can be compared with those obtained by flow-cytometry, this, we believe, will potentially aid research on the GPCR field and many other membrane expressed receptors.

Altogether, these results demonstrate the reliability of this novel bioassay in quantifying the CB1 receptor expression on the cell surface.

## Supplementary information

Supplementary Information

## Data Availability

The datasets generated during the current study are available from the corresponding author on request. The plasmid (*GLuc-FLAG-CNR1*) will be available from Addgene.
