# Supplementary material for: A novel bioassay for quantification of surface Cannabinoid receptor 1 expression
Source: Sci Rep. 2020 Oct 23;10:18191. doi: 10.1038/s41598-020-75331-y (PMC7584592; doi:10.1038/s41598-020-75331-y)
Supplement: Supplementary file 1 — Supplementary Information [file 41598_2020_75331_MOESM1_ESM.docx]

**Supporting Information**

**A novel bioassay for quantification of surface Cannabinoid receptor 1 expression**

Ismael Rodríguez-Rodríguez^§1^, Joanna Kalafut^§2^, Arkadiusz Czerwonka^3^, Adolfo Rivero-Muller^2,4^*

§Equal contribution

*Correspondence: [a.rivero@umlub.pl](mailto:a.rivero@umlub.pl)

^1^Department of Organic Chemistry, Faculty of Chemistry, Jagiellonian University, Krakow, Poland
^2^Department of Biochemistry and Molecular Biology, Medical University of Lublin, Poland
^3^ Department of Virology and Immunology, Faculty of Biology and Biotechnology, Maria Curie-Skłodowska University, Lublin, Poland.


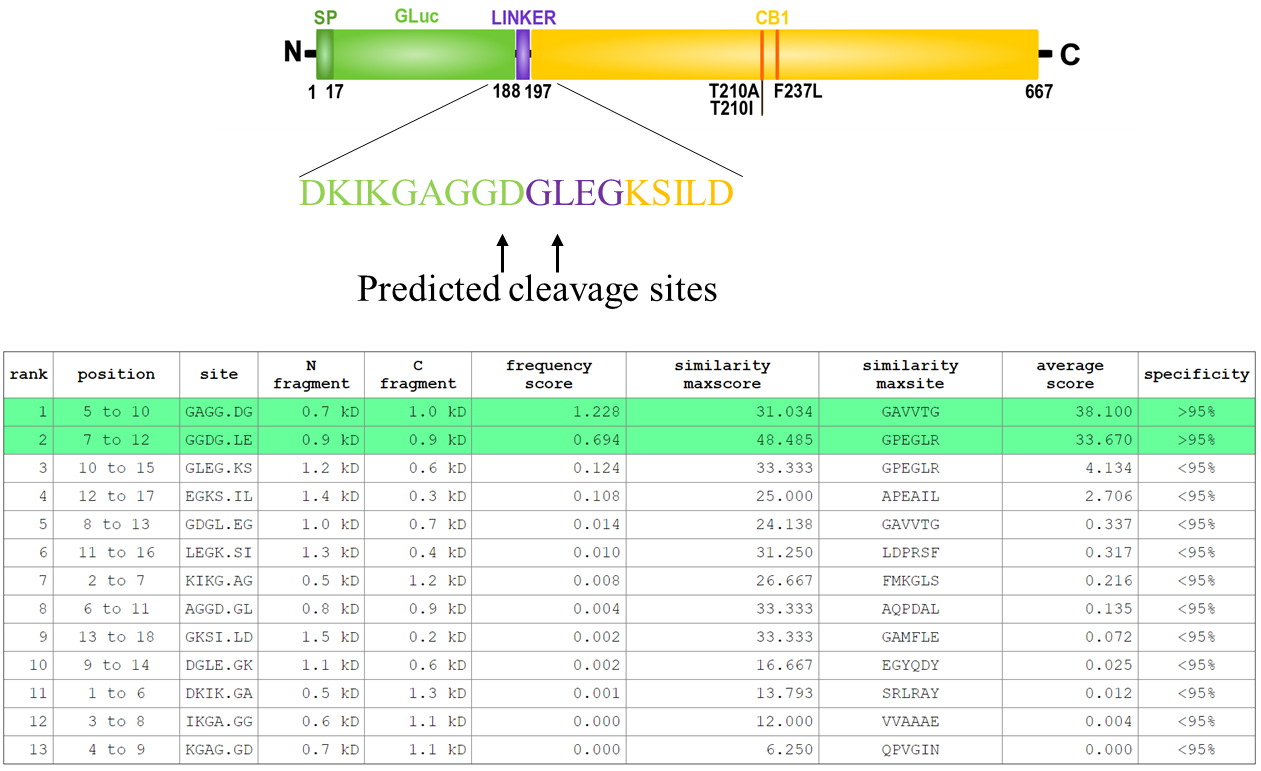


**Supplementary Figure 1**. The sequence DKIKGAGGDGLEGKSILD appears to have two potential cleavage sites that could disrupt the generated chimeric protein GLuc-GLUG-CB1, as predicted by the software tool “SitePrediction” ^1^.


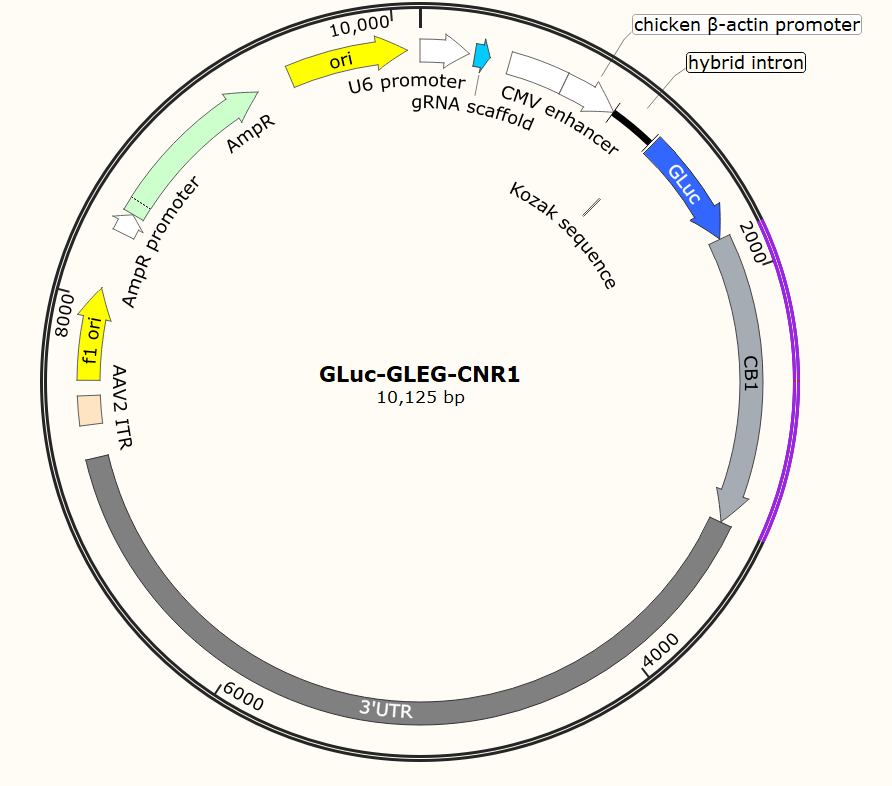


**Supplementary Fig. 2.** Plasmid *GLuc-GLEG-CNR1*

**Nucleic acids sequence of the construct *GLuc–GLEG–CNR1* (10,125 bp)**

**GLuc**

**LINKER**

**CB1**

**3’UTR**

GAGGGCCTATTTCCCATGATTCCTTCATATTTGCATATACGATACAAGGCTGTTAGAGAGATAATTGGAATTAATTTGACTGTAAACACAAAGATATTAGTACAAAATACGTGACGTAGAAAGTAATAATTTCTTGGGTAGTTTGCAGTTTTAAAATTATGTTTTAAAATGGACTATCATATGCTTACCGTAACTTGAAAGTATTTCGATTTCTTGGCTTTATATATCTTGTGGAAAGGACGAAACACCGGGTCTTCGAGAAGACCTGTTTTAGAGCTAGAAATAGCAAGTTAAAATAAGGCTAGTCCGTTATCAACTTGAAAAAGTGGCACCGAGTCGGTGCTTTTTTGTTTTAGAGCTAGAAATAGCAAGTTAAAATAAGGCTAGTCCGTTTTTAGCGCGTGCGCCAATTCTGCAGACAAATGGCTCTAGAGGTACCCGTTACATAACTTACGGTAAATGGCCCGCCTGGCTGACCGCCCAACGACCCCCGCCCATTGACGTCAATAGTAACGCCAATAGGGACTTTCCATTGACGTCAATGGGTGGAGTATTTACGGTAAACTGCCCACTTGGCAGTACATCAAGTGTATCATATGCCAAGTACGCCCCCTATTGACGTCAATGACGGTAAATGGCCCGCCTGGCATTGTGCCCAGTACATGACCTTATGGGACTTTCCTACTTGGCAGTACATCTACGTATTAGTCATCGCTATTACCATGGTCGAGGTGAGCCCCACGTTCTGCTTCACTCTCCCCATCTCCCCCCCCTCCCCACCCCCAATTTTGTATTTATTTATTTTTTAATTATTTTGTGCAGCGATGGGGGCGGGGGGGGGGGGGGGGCGCGCGCCAGGCGGGGCGGGGCGGGGCGAGGGGCGGGGCGGGGCGAGGCGGAGAGGTGCGGCGGCAGCCAATCAGAGCGGCGCGCTCCGAAAGTTTCCTTTTATGGCGAGGCGGCGGCGGCGGCGGCCCTATAAAAAGCGAAGCGCGCGGCGGGCGGGAGTCGCTGCGACGCTGCCTTCGCCCCGTGCCCCGCTCCGCCGCCGCCTCGCGCCGCCCGCCCCGGCTCTGACTGACCGCGTTACTCCCACAGGTGAGCGGGCGGGACGGCCCTTCTCCTCCGGGCTGTAATTAGCTGAGCAAGAGGTAAGGGTTTAAGGGATGGTTGGTTGGTGGGGTATTAATGTTTAATTACCTGGAGCACCTGCCTGAAATCACTTTTTTTCAGGTTGGACCGGTGCCACCATGGGAGTCAAAGTTCTGTTTGCCCTGATCTGCATCGCTGTGGCCGAGGCCAAGCCCACCGAGAACAACGAAGACTTCAACATCGTGGCCGTGGCCAGCAACTTCGCGACCACGGATCTCGATGCTGACCGCGGGAAGTTGCCCGGCAAGAAGCTGCCGCTGGAGGTGCTCAAAGAGATGGAAGCCAATGCCCGGAAAGCTGGCTGCACCAGGGGCTGTCTGATCTGCCTGTCCCACATCAAGTGCACGCCCAAGATGAAGAAGTTCATCCCAGGACGCTGCCACACCTACGAAGGCGACAAAGAGTCCGCACAGGGCGGCATAGGCGAGGCGATCGTCGACATTCCTGAGATTCCTGGGTTCAAGGACTTGGAGCCCATGGAGCAGTTCATCGCACAGGTCGATCTGTGTGTGGACTGCACAACTGGCTGCCTCAAAGGGCTTGCCAACGTGCAGTGTTCTGACCTGCTCAAGAAGTGGCTGCCGCAACGCTGTGCGACCTTTGCCAGCAAGATCCAGGGCCAGGTGGACAAGATCAAGGGGGCCGGTGGTGACGGACTCGAGGGTAAGTCGATCCTAGATGGCCTTGCAGATACCACCTTCCGCACCATCACCACTGACCTCCTGTACGTGGGCTCAAATGACATTCAGTACGAAGACATCAAAGGTGACATGGCATCCAAATTAGGGTACTTCCCACAGAAATTCCCTTTAACTTCCTTTAGGGGAAGTCCCTTCCAAGAGAAGATGACTGCGGGAGACAACCCCCAGCTAGTCCCAGCAGACCAGGTGAACATTACAGAATTTTACAACAAGTCTCTCTCGTCCTTCAAGGAGAATGAGGAGAACATCCAGTGTGGGGAGAACTTCATGGACATAGAGTGTTTCATGGTCCTGAACCCCAGCCAGCAGCTGGCCATTGCAGTCCTGTCCCTCACGCTGGGCACCTTCACGGTCCTGGAGAACCTCCTGGTGCTGTGCGTCATCCTCCACTCCCGCAGCCTCCGCTGCAGGCCTTCCTACCACTTCATCGGCAGCCTGGCGGTGGCAGACCTCCTGGGGAGTGTCATTTTTGTCTACAGCTTCATTGACTTCCACGTGTTCCACCGCAAAGATAGCCGCAACGTGTTTCTGTTCAAACTGGGTGGGGTCACGGCCTCCTTCACTGCCTCCGTGGGCAGCCTGTTCCTCACAGCCATCGACAGGTACATATCCATTCACAGGCCCCTGGCCTATAAGAGGATTGTCACCAGGCCCAAGGCCGTGGTGGCGTTTTGCCTGATGTGGACCATAGCCATTGTGATCGCCGTGCTGCCTCTCCTGGGCTGGAACTGCGAGAAACTGCAATCTGTTTGCTCAGACATTTTCCCACACATTGATGAAACCTACCTGATGTTCTGGATCGGGGTCACCAGCGTACTGCTTCTGTTCATCGTGTATGCGTACATGTATATTCTCTGGAAGGCTCACAGCCACGCCGTCCGCATGATTCAGCGTGGCACCCAGAAGAGCATCATCATCCACACGTCTGAGGATGGGAAGGTACAGGTGACCCGGCCAGACCAAGCCCGCATGGACATTAGGTTAGCCAAGACCCTGGTCCTGATCCTGGTGGTGTTGATCATCTGCTGGGGCCCTCTGCTTGCAATCATGGTGTATGATGTCTTTGGGAAGATGAACAAGCTCATTAAGACGGTGTTTGCATTCTGCAGTATGCTCTGCCTGCTGAACTCCACCGTGAACCCCATCATCTATGCTCTGAGGAGTAAGGACCTGCGACACGCTTTCCGGAGCATGTTTCCCTCTTGTGAAGGCACTGCGCAGCCTCTGGATAACAGCATGGGGGACTCGGACTGCCTGCACAAACACGCAAACAATGCAGCCAGTGTTCACAGGGCCGCAGAAAGCTGCATCAAGAGCACGGTCAAGATTGCCAAGGTAACCATGTCTGTGTCCACAGACACGTCTGCCGAGGCTCTGTGAGCCTGATGCCTCCCTGGCAGCACAGGAAAAGAATTTTTTTTTTTAAGCTCAAAATCTAGAAGAGTCTATTGTCTCCTTGGTTATATTTTTTTAACTTTACCATGCTCAATGAAAAGGTGATTGTCACCATGATCACTTATCAGTTTGCTAATGTTTCCATAGTTTAGGTACTCAAACTCCATTCTCCAGGGGTTTACAGTGAAGAAAGCCTGTTGTTTAAGTGACTGAACGATCCTTCAAAGTCTCAATGAAATAGGAGGGAAACCTTTGGCTACACAATTGGAAGTCTAAGAACCCATGGAAAAATGCCATCAAATGAATAATGCCTTTGTAACCACAACTTTCACTATAATGTGAAATGTAACTGTCCGTAGTATCAGAGATGTCCATTTTTACAAGTTATAGTACTAGAGATATTTTGTAAAATGTATTATGTCCTGTGAGATGTGTATCAGTGTTTATGTGCTATTAATATTTGTTTAGTTCAGCAAAACTGAAAGGTAGACTTTTATGAGAACAATGGACAAGCAGTGGATACGTGTCAATGTGTGCACTTTTTTTCTATATTATTGCCCATGATATAACTTTAGAAATAAACCTTAATATTTCTTCAAATATCTCTATTTAATTTTGACACTGAAATAACCGTAAAGGTTTATTTTTCTGTTACCTCAACAAGAAGAATTTGAAGACTTCAAAATATTGAGCAGAATTCATTCATACTTAAAAATTTATTAGCCCTGCATTTTCATAGGAAGACACATTATCTTCTGGACTATAGCTGTTCTAATGGATTATAATCAGAATGGAAGAGAGAAAGCATATTGACTTTTTTTGAGCGACATCTCTGACTTTCTTTAGTCTTTAGCTATTACTGGATCTCTTAAGACAGCATGTGTTAATCTTAATGTATATCGTTATCACTGTGCAGTTGCTGTTTACTTGAATAGTATTGTGTTCCTATATTCCAGGTTTAAGTAGATTTCATGCCTGGGTGGCCAAACAACAGTCTTCATTTTTTTTAATTGAAAAGAAGTAGTGTCTGGATCAGTAAAATTATACTGTGTGTGAGTGTGAATATAAATGTGTGTATGTGTGTTTCTGTCCTGTAACTGTTACAGTAATGTCATAAAGTGAGAAAACTGTGACCAAGTATAAACTTTTACCACTTGCTGCACTCTTGCACATGGATTCAGTTTCTAAAATTGAGTTCTTCCTGTAATCTTGTTGATAAAAATACTGACTCCAACCATTCAAAAATTTCACCCCATCCCTCCTTAAGAGATTGGATCAAGTATTACTAAATTGACCTTTAGGTATTACACAAGACCAGTGCTTAGCAAAAAATAATGACAGGCATCCAAGGAAGGGATGTATTTGTAGTGTTATTGCCAGGAAAGGAGAGTACTTTGGTTTCTGAGCACCGAATATTGAGCAATATGTCAGTCACTAAAAGGAAGACAGTTCTACAGAAAAACAATGGTAACATTTTTCAATAGCGTGTGTAGATAGTATGCACTATATACATCACGTTAAAGTAGGACTATCACACCCAGCCCATGTGGCTAAAAAAGCTGAATCAGACAGTGGATGAGACACACAACGGCAGTGAAGAACCGATACACTTGGCATTGACGTCTAGCTATGCTGTATCTGTGCTTTGCCCACATGCCCTTGGTGACAGCTGAGCACCCAGCTCTGTCTTGGTAGGTTTGGGCTAAGGAACAAATCTCTCCTTTGCTCGTGGTTAGCAAGATACACTCAAGCATGAAGATAAACACAGCTGCTTTCTTCTTACACCCCGGTCTCATGCTCCTTAATGGCGCCATGGGTGCTTGTTGGGCCTTTTTCCAGTAAGGAATGATATTGCTGAAGAATCTACTTAACCCTGACAAATTTTAATTATAATCTCTTCTTATACAGATAAAACATGACTCCTACAAGGCCCCAAGGTTTACATAGTCTGAAGTGAAGTACAGAGCTGGCATCTATCTGGTGATTTCTAGCTCTCGAGATACCCAAGCAGCCTGATGGGGCAGTTCCCCTTCTTACGGTTCACGCTCTAAGGCAGGATGTGGCTTATGAGATACTTTGCATTGTCTGTCTGCACACCTTGAATCTGCCTGCTGGCTCCCTTACTTTACCTCTCTGTCATGTGCAGATGAAGGCTCAGGGTGCTAGAGGATTAGTAAGATCTCTTTCTAAAGACAGGAGAGATTATTTACAAGAAGAACTCACCAGGGTTTAGTTTGCATTTAAGAATTGCCAGTCTTTTGTCCTGCATCATCTTGAACATTAATCCACATGTTTCAGAGCTCACCAGGCAGTACCAATGCTCTTTTCACAGCTATGAAGAGCTAGAGAAATTCTTGTTATGGTAGAAAAATTTCACGATTCATTTTTGAAACTGCATTTGTGCGTATGCAGTGTAGATTTTATAGTGTGTTGTGCTTTCAAGATCTAAATCATATATAATAAATTAAGGGACAATGGGGCTGACAGCACTAAACTTGGTGCTTATTGATATTCTAAGAAATATCTGTGAAATATCATCACGTATGTTATACAACCTTCATTTAAAAAGGTTTAAAACTAGTTAGATTCACTTTGACACTTTTCATATCATTTCTTAACCCAAGTGACGAAAACATTGTCCCCAATGAATATACTCATTAGAATTACCATTTGTTAATATCACTCATTAATTAACCCCATAATTAGATCCATTAATTTAAATGATTTAAATTTAAGTAAGTTTTATAAGGTCTGACATCAGAGGTATCTTACTTTCCTCTGAGGATGATGTACTTGCCCTGACCATGCATTTTACCATCACACATGTTCAGAAAGGGCCAAATTCCCAACCTGCTCATTTTTTTTTTATCAGAGTCATGATGAATCAGTCCTAGAATGTTTCATTTGCACAAGTAGGGCTGCCTCCAAGAGGAACCTCTGATTTATTTTGTATGAAATATATGTGAAAGGATATGAATCTGAGAGATGCTGTAGACATCTGTCCTACACTTGAGATGATTTCCAAGCCTCTCTGGCACTTTGAGTTAAGTCTATCTGGTATTAAATGCCAAGGACCTTTTGCTGCCTAAATCCACTCTGCAGGAAATAGGCCCAACCACCAGATGAGAATTAGGCCCTGGATGAGTAGCGCTATAGTTACTGTCCTGTTGATTAATTTCTGCCATTTCATGTCCATAAAAGAGACCACCCATATCATGCACACAATTAGATTTCTCACACTCTAACTGTATATTTGTATGATATTTTAAAATCTCCTAAATGCTGGGCAATGGCTATTAACAATTAATTGTCTTGCACTGGCCTTCTGATGAAATGTTAACAATGCCTATTGTAATATAGAAAAAAACATTCTATCTACTGATTTGGGCTGAATGTATGTAAATAGGTTTCTAAAAAGTCAGATGTTTGAGCAGTGGCCTACAAATCAGTAATTTTCGGATGGGAGAGTTTCTTTACATTGCCGTGGCATCTTAAAAGCTATCTTCATGTAAATTGACTGTACTAGGCCTACTGGGGATCAGAGTTCCCAAGAAAGGAAACCTTTTCTTGTATCTGGATTCAAATTTATTTCCAATGTTTCAAGCGGGAAACATGACTCTTTATTGTCTGTAAATCTAACATTATTACTTTTCCTCTTAGAAGAATATTGTATTGTTAGATGTTTGTTGAGCTGGTAACATCGTTGCAACCACTGCAATATCTTCGTTAGTAATCTGTATAATACTTTGTATACAAGTACTGGTAAGATTGTTATTAAATGTAGCTTCAGTCATTAAATTACTATAGCAAAGTAGTACTTCTTCTGTAATATTTACAATGTATTAAGCCCACAGTATATTTTATTTCAATGTAATTAAACTGTTAACTTATTCAAAGAGAAAACATCTCATCATGTCTATTGTCCAAAGTTACCTGGAATCAAATAAAAATTCTAGATTACCATGAAGAACATAAAATGCCTTTGAACTCTGCCTTATTTCACAGTCTGATGGCAAAAAATGAGGAAATTGCATCGCATTGTCTGAGTAGGTGTCATTCTATTCTGGGGGGTGGGGTGGGGCAGGACAGCAAGGGGGAGGATTGGGAAGAGAATAGCAGGCATGCTGGGGAGCGGCCGCAGGAACCCCTAGTGATGGAGTTGGCCACTCCCTCTCTGCGCGCTCGCTCGCTCACTGAGGCCGGGCGACCAAAGGTCGCCCGACGCCCGGGCTTTGCCCGGGCGGCCTCAGTGAGCGAGCGAGCGCGCAGCTGCCTGCAGGGGCGCCTGATGCGGTATTTTCTCCTTACGCATCTGTGCGGTATTTCACACCGCATACGTCAAAGCAACCATAGTACGCGCCCTGTAGCGGCGCATTAAGCGCGGCGGGTGTGGTGGTTACGCGCAGCGTGACCGCTACACTTGCCAGCGCCCTAGCGCCCGCTCCTTTCGCTTTCTTCCCTTCCTTTCTCGCCACGTTCGCCGGCTTTCCCCGTCAAGCTCTAAATCGGGGGCTCCCTTTAGGGTTCCGATTTAGTGCTTTACGGCACCTCGACCCCAAAAAACTTGATTTGGGTGATGGTTCACGTAGTGGGCCATCGCCCTGATAGACGGTTTTTCGCCCTTTGACGTTGGAGTCCACGTTCTTTAATAGTGGACTCTTGTTCCAAACTGGAACAACACTCAACCCTATCTCGGGCTATTCTTTTGATTTATAAGGGATTTTGCCGATTTCGGCCTATTGGTTAAAAAATGAGCTGATTTAACAAAAATTTAACGCGAATTTTAACAAAATATTAACGTTTACAATTTTATGGTGCACTCTCAGTACAATCTGCTCTGATGCCGCATAGTTAAGCCAGCCCCGACACCCGCCAACACCCGCTGACGCGCCCTGACGGGCTTGTCTGCTCCCGGCATCCGCTTACAGACAAGCTGTGACCGTCTCCGGGAGCTGCATGTGTCAGAGGTTTTCACCGTCATCACCGAAACGCGCGAGACGAAAGGGCCTCGTGATACGCCTATTTTTATAGGTTAATGTCATGATAATAATGGTTTCTTAGACGTCAGGTGGCACTTTTCGGGGAAATGTGCGCGGAACCCCTATTTGTTTATTTTTCTAAATACATTCAAATATGTATCCGCTCATGAGACAATAACCCTGATAAATGCTTCAATAATATTGAAAAAGGAAGAGTATGAGTATTCAACATTTCCGTGTCGCCCTTATTCCCTTTTTTGCGGCATTTTGCCTTCCTGTTTTTGCTCACCCAGAAACGCTGGTGAAAGTAAAAGATGCTGAAGATCAGTTGGGTGCACGAGTGGGTTACATCGAACTGGATCTCAACAGCGGTAAGATCCTTGAGAGTTTTCGCCCCGAAGAACGTTTTCCAATGATGAGCACTTTTAAAGTTCTGCTATGTGGCGCGGTATTATCCCGTATTGACGCCGGGCAAGAGCAACTCGGTCGCCGCATACACTATTCTCAGAATGACTTGGTTGAGTACTCACCAGTCACAGAAAAGCATCTTACGGATGGCATGACAGTAAGAGAATTATGCAGTGCTGCCATAACCATGAGTGATAACACTGCGGCCAACTTACTTCTGACAACGATCGGAGGACCGAAGGAGCTAACCGCTTTTTTGCACAACATGGGGGATCATGTAACTCGCCTTGATCGTTGGGAACCGGAGCTGAATGAAGCCATACCAAACGACGAGCGTGACACCACGATGCCTGTAGCAATGGCAACAACGTTGCGCAAACTATTAACTGGCGAACTACTTACTCTAGCTTCCCGGCAACAATTAATAGACTGGATGGAGGCGGATAAAGTTGCAGGACCACTTCTGCGCTCGGCCCTTCCGGCTGGCTGGTTTATTGCTGATAAATCTGGAGCCGGTGAGCGTGGAAGCCGCGGTATCATTGCAGCACTGGGGCCAGATGGTAAGCCCTCCCGTATCGTAGTTATCTACACGACGGGGAGTCAGGCAACTATGGATGAACGAAATAGACAGATCGCTGAGATAGGTGCCTCACTGATTAAGCATTGGTAACTGTCAGACCAAGTTTACTCATATATACTTTAGATTGATTTAAAACTTCATTTTTAATTTAAAAGGATCTAGGTGAAGATCCTTTTTGATAATCTCATGACCAAAATCCCTTAACGTGAGTTTTCGTTCCACTGAGCGTCAGACCCCGTAGAAAAGATCAAAGGATCTTCTTGAGATCCTTTTTTTCTGCGCGTAATCTGCTGCTTGCAAACAAAAAAACCACCGCTACCAGCGGTGGTTTGTTTGCCGGATCAAGAGCTACCAACTCTTTTTCCGAAGGTAACTGGCTTCAGCAGAGCGCAGATACCAAATACTGTCCTTCTAGTGTAGCCGTAGTTAGGCCACCACTTCAAGAACTCTGTAGCACCGCCTACATACCTCGCTCTGCTAATCCTGTTACCAGTGGCTGCTGCCAGTGGCGATAAGTCGTGTCTTACCGGGTTGGACTCAAGACGATAGTTACCGGATAAGGCGCAGCGGTCGGGCTGAACGGGGGGTTCGTGCACACAGCCCAGCTTGGAGCGAACGACCTACACCGAACTGAGATACCTACAGCGTGAGCTATGAGAAAGCGCCACGCTTCCCGAAGGGAGAAAGGCGGACAGGTATCCGGTAAGCGGCAGGGTCGGAACAGGAGAGCGCACGAGGGAGCTTCCAGGGGGAAACGCCTGGTATCTTTATAGTCCTGTCGGGTTTCGCCACCTCTGACTTGAGCGTCGATTTTTGTGATGCTCGTCAGGGGGGCGGAGCCTATGGAAAAACGCCAGCAACGCGGCCTTTTTACGGTTCCTGGCCTTTTGCTGGCCTTTTGCTCACATGT

**Amino acid sequence of the chimeric proteins GLuc–GLEG–CB1 (660 AA)**

MGVKVLFALICIAVAEAKPTENNEDFNIVAVASNFATTDLDADRGKLPGKKLPLEVLKEMEANARKAGCTRGCLICLSHIKCTPKMKKFIPGRCHTYEGDKESAQGGIGEAIVDIPEIPGFKDLEPMEQFIAQVDLCVDCTTGCLKGLANVQCSDLLKKWLPQRCATFASKIQGQVDKIKGAGGDGLEGKSILDGLADTTFRTITTDLLYVGSNDIQYEDIKGDMASKLGYFPQKFPLTSFRGSPFQEKMTAGDNPQLVPADQVNITEFYNKSLSSFKENEENIQCGENFMDIECFMVLNPSQQLAIAVLSLTLGTFTVLENLLVLCVILHSRSLRCRPSYHFIGSLAVADLLGSVIFVYSFIDFHVFHRKDSRNVFLFKLGGVTASFTASVGSLFLTAIDRYISIHRPLAYKRIVTRPKAVVAFCLMWTIAIVIAVLPLLGWNCEKLQSVCSDIFPHIDETYLMFWIGVTSVLLLFIVYAYMYILWKAHSHAVRMIQRGTQKSIIIHTSEDGKVQVTRPDQARMDIRLAKTLVLILVVLIICWGPLLAIMVYDVFGKMNKLIKTVFAFCSMLCLLNSTVNPIIYALRSKDLRHAFRSMFPSCEGTAQPLDNSMGDSDCLHKHANNAASVHRAAESCIKSTVKIAKVTMSVSTDTSAEAL


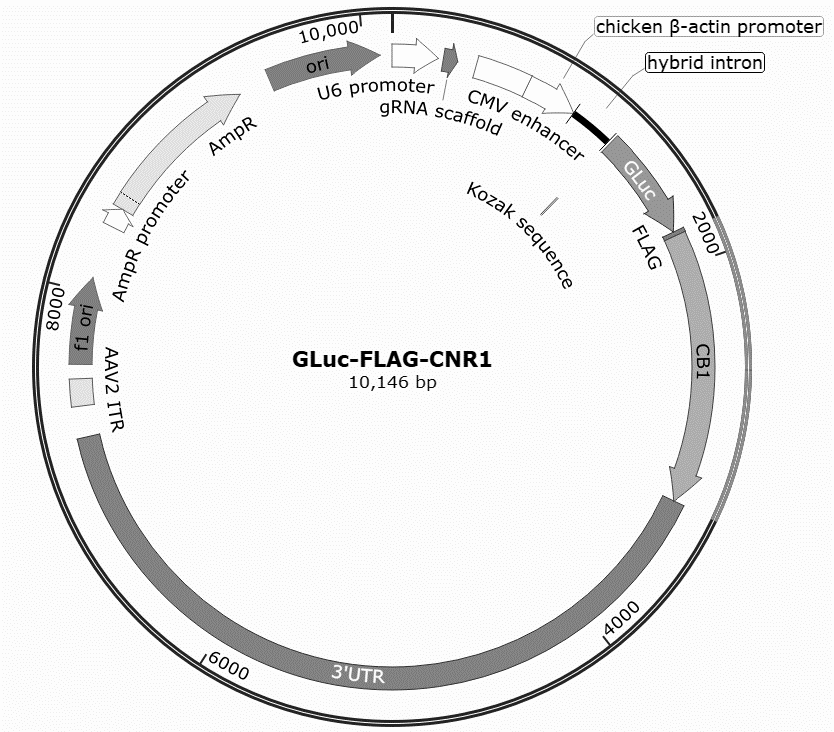


**Supplementary Fig. 3.** Plasmid *GLuc-FLAG-CNR1*

**Nucleic acid sequence of the construct *GLuc–FLAG–CNR1* (10,146 bp)**

**GLuc**

**LINKER**

**CB1**

**3’UTR**

GAGGGCCTATTTCCCATGATTCCTTCATATTTGCATATACGATACAAGGCTGTTAGAGAGATAATTGGAATTAATTTGACTGTAAACACAAAGATATTAGTACAAAATACGTGACGTAGAAAGTAATAATTTCTTGGGTAGTTTGCAGTTTTAAAATTATGTTTTAAAATGGACTATCATATGCTTACCGTAACTTGAAAGTATTTCGATTTCTTGGCTTTATATATCTTGTGGAAAGGACGAAACACCGGGTCTTCGAGAAGACCTGTTTTAGAGCTAGAAATAGCAAGTTAAAATAAGGCTAGTCCGTTATCAACTTGAAAAAGTGGCACCGAGTCGGTGCTTTTTTGTTTTAGAGCTAGAAATAGCAAGTTAAAATAAGGCTAGTCCGTTTTTAGCGCGTGCGCCAATTCTGCAGACAAATGGCTCTAGAGGTACCCGTTACATAACTTACGGTAAATGGCCCGCCTGGCTGACCGCCCAACGACCCCCGCCCATTGACGTCAATAGTAACGCCAATAGGGACTTTCCATTGACGTCAATGGGTGGAGTATTTACGGTAAACTGCCCACTTGGCAGTACATCAAGTGTATCATATGCCAAGTACGCCCCCTATTGACGTCAATGACGGTAAATGGCCCGCCTGGCATTGTGCCCAGTACATGACCTTATGGGACTTTCCTACTTGGCAGTACATCTACGTATTAGTCATCGCTATTACCATGGTCGAGGTGAGCCCCACGTTCTGCTTCACTCTCCCCATCTCCCCCCCCTCCCCACCCCCAATTTTGTATTTATTTATTTTTTAATTATTTTGTGCAGCGATGGGGGCGGGGGGGGGGGGGGGGCGCGCGCCAGGCGGGGCGGGGCGGGGCGAGGGGCGGGGCGGGGCGAGGCGGAGAGGTGCGGCGGCAGCCAATCAGAGCGGCGCGCTCCGAAAGTTTCCTTTTATGGCGAGGCGGCGGCGGCGGCGGCCCTATAAAAAGCGAAGCGCGCGGCGGGCGGGAGTCGCTGCGACGCTGCCTTCGCCCCGTGCCCCGCTCCGCCGCCGCCTCGCGCCGCCCGCCCCGGCTCTGACTGACCGCGTTACTCCCACAGGTGAGCGGGCGGGACGGCCCTTCTCCTCCGGGCTGTAATTAGCTGAGCAAGAGGTAAGGGTTTAAGGGATGGTTGGTTGGTGGGGTATTAATGTTTAATTACCTGGAGCACCTGCCTGAAATCACTTTTTTTCAGGTTGGACCGGTGCCACCATGGGAGTCAAAGTTCTGTTTGCCCTGATCTGCATCGCTGTGGCCGAGGCCAAGCCCACCGAGAACAACGAAGACTTCAACATCGTGGCCGTGGCCAGCAACTTCGCGACCACGGATCTCGATGCTGACCGCGGGAAGTTGCCCGGCAAGAAGCTGCCGCTGGAGGTGCTCAAAGAGATGGAAGCCAATGCCCGGAAAGCTGGCTGCACCAGGGGCTGTCTGATCTGCCTGTCCCACATCAAGTGCACGCCCAAGATGAAGAAGTTCATCCCAGGACGCTGCCACACCTACGAAGGCGACAAAGAGTCCGCACAGGGCGGCATAGGCGAGGCGATCGTCGACATTCCTGAGATTCCTGGGTTCAAGGACTTGGAGCCCATGGAGCAGTTCATCGCACAGGTCGATCTGTGTGTGGACTGCACAACTGGCTGCCTCAAAGGGCTTGCCAACGTGCAGTGTTCTGACCTGCTCAAGAAGTGGCTGCCGCAACGCTGTGCGACCTTTGCCAGCAAGATCCAGGGCCAGGTGGACAAGATCAAGGGGGCCGGTGGTGACGGACTCGAGGATTACAAGGATGACGACGATAAGAAGTCGATCCTAGATGGCCTTGCAGATACCACCTTCCGCACCATCACCACTGACCTCCTGTACGTGGGCTCAAATGACATTCAGTACGAAGACATCAAAGGTGACATGGCATCCAAATTAGGGTACTTCCCACAGAAATTCCCTTTAACTTCCTTTAGGGGAAGTCCCTTCCAAGAGAAGATGACTGCGGGAGACAACCCCCAGCTAGTCCCAGCAGACCAGGTGAACATTACAGAATTTTACAACAAGTCTCTCTCGTCCTTCAAGGAGAATGAGGAGAACATCCAGTGTGGGGAGAACTTCATGGACATAGAGTGTTTCATGGTCCTGAACCCCAGCCAGCAGCTGGCCATTGCAGTCCTGTCCCTCACGCTGGGCACCTTCACGGTCCTGGAGAACCTCCTGGTGCTGTGCGTCATCCTCCACTCCCGCAGCCTCCGCTGCAGGCCTTCCTACCACTTCATCGGCAGCCTGGCGGTGGCAGACCTCCTGGGGAGTGTCATTTTTGTCTACAGCTTCATTGACTTCCACGTGTTCCACCGCAAAGATAGCCGCAACGTGTTTCTGTTCAAACTGGGTGGGGTCACGGCCTCCTTCACTGCCTCCGTGGGCAGCCTGTTCCTCACAGCCATCGACAGGTACATATCCATTCACAGGCCCCTGGCCTATAAGAGGATTGTCACCAGGCCCAAGGCCGTGGTGGCGTTTTGCCTGATGTGGACCATAGCCATTGTGATCGCCGTGCTGCCTCTCCTGGGCTGGAACTGCGAGAAACTGCAATCTGTTTGCTCAGACATTTTCCCACACATTGATGAAACCTACCTGATGTTCTGGATCGGGGTCACCAGCGTACTGCTTCTGTTCATCGTGTATGCGTACATGTATATTCTCTGGAAGGCTCACAGCCACGCCGTCCGCATGATTCAGCGTGGCACCCAGAAGAGCATCATCATCCACACGTCTGAGGATGGGAAGGTACAGGTGACCCGGCCAGACCAAGCCCGCATGGACATTAGGTTAGCCAAGACCCTGGTCCTGATCCTGGTGGTGTTGATCATCTGCTGGGGCCCTCTGCTTGCAATCATGGTGTATGATGTCTTTGGGAAGATGAACAAGCTCATTAAGACGGTGTTTGCATTCTGCAGTATGCTCTGCCTGCTGAACTCCACCGTGAACCCCATCATCTATGCTCTGAGGAGTAAGGACCTGCGACACGCTTTCCGGAGCATGTTTCCCTCTTGTGAAGGCACTGCGCAGCCTCTGGATAACAGCATGGGGGACTCGGACTGCCTGCACAAACACGCAAACAATGCAGCCAGTGTTCACAGGGCCGCAGAAAGCTGCATCAAGAGCACGGTCAAGATTGCCAAGGTAACCATGTCTGTGTCCACAGACACGTCTGCCGAGGCTCTGTGAGCCTGATGCCTCCCTGGCAGCACAGGAAAAGAATTTTTTTTTTTAAGCTCAAAATCTAGAAGAGTCTATTGTCTCCTTGGTTATATTTTTTTAACTTTACCATGCTCAATGAAAAGGTGATTGTCACCATGATCACTTATCAGTTTGCTAATGTTTCCATAGTTTAGGTACTCAAACTCCATTCTCCAGGGGTTTACAGTGAAGAAAGCCTGTTGTTTAAGTGACTGAACGATCCTTCAAAGTCTCAATGAAATAGGAGGGAAACCTTTGGCTACACAATTGGAAGTCTAAGAACCCATGGAAAAATGCCATCAAATGAATAATGCCTTTGTAACCACAACTTTCACTATAATGTGAAATGTAACTGTCCGTAGTATCAGAGATGTCCATTTTTACAAGTTATAGTACTAGAGATATTTTGTAAAATGTATTATGTCCTGTGAGATGTGTATCAGTGTTTATGTGCTATTAATATTTGTTTAGTTCAGCAAAACTGAAAGGTAGACTTTTATGAGAACAATGGACAAGCAGTGGATACGTGTCAATGTGTGCACTTTTTTTCTATATTATTGCCCATGATATAACTTTAGAAATAAACCTTAATATTTCTTCAAATATCTCTATTTAATTTTGACACTGAAATAACCGTAAAGGTTTATTTTTCTGTTACCTCAACAAGAAGAATTTGAAGACTTCAAAATATTGAGCAGAATTCATTCATACTTAAAAATTTATTAGCCCTGCATTTTCATAGGAAGACACATTATCTTCTGGACTATAGCTGTTCTAATGGATTATAATCAGAATGGAAGAGAGAAAGCATATTGACTTTTTTTGAGCGACATCTCTGACTTTCTTTAGTCTTTAGCTATTACTGGATCTCTTAAGACAGCATGTGTTAATCTTAATGTATATCGTTATCACTGTGCAGTTGCTGTTTACTTGAATAGTATTGTGTTCCTATATTCCAGGTTTAAGTAGATTTCATGCCTGGGTGGCCAAACAACAGTCTTCATTTTTTTTAATTGAAAAGAAGTAGTGTCTGGATCAGTAAAATTATACTGTGTGTGAGTGTGAATATAAATGTGTGTATGTGTGTTTCTGTCCTGTAACTGTTACAGTAATGTCATAAAGTGAGAAAACTGTGACCAAGTATAAACTTTTACCACTTGCTGCACTCTTGCACATGGATTCAGTTTCTAAAATTGAGTTCTTCCTGTAATCTTGTTGATAAAAATACTGACTCCAACCATTCAAAAATTTCACCCCATCCCTCCTTAAGAGATTGGATCAAGTATTACTAAATTGACCTTTAGGTATTACACAAGACCAGTGCTTAGCAAAAAATAATGACAGGCATCCAAGGAAGGGATGTATTTGTAGTGTTATTGCCAGGAAAGGAGAGTACTTTGGTTTCTGAGCACCGAATATTGAGCAATATGTCAGTCACTAAAAGGAAGACAGTTCTACAGAAAAACAATGGTAACATTTTTCAATAGCGTGTGTAGATAGTATGCACTATATACATCACGTTAAAGTAGGACTATCACACCCAGCCCATGTGGCTAAAAAAGCTGAATCAGACAGTGGATGAGACACACAACGGCAGTGAAGAACCGATACACTTGGCATTGACGTCTAGCTATGCTGTATCTGTGCTTTGCCCACATGCCCTTGGTGACAGCTGAGCACCCAGCTCTGTCTTGGTAGGTTTGGGCTAAGGAACAAATCTCTCCTTTGCTCGTGGTTAGCAAGATACACTCAAGCATGAAGATAAACACAGCTGCTTTCTTCTTACACCCCGGTCTCATGCTCCTTAATGGCGCCATGGGTGCTTGTTGGGCCTTTTTCCAGTAAGGAATGATATTGCTGAAGAATCTACTTAACCCTGACAAATTTTAATTATAATCTCTTCTTATACAGATAAAACATGACTCCTACAAGGCCCCAAGGTTTACATAGTCTGAAGTGAAGTACAGAGCTGGCATCTATCTGGTGATTTCTAGCTCTCGAGATACCCAAGCAGCCTGATGGGGCAGTTCCCCTTCTTACGGTTCACGCTCTAAGGCAGGATGTGGCTTATGAGATACTTTGCATTGTCTGTCTGCACACCTTGAATCTGCCTGCTGGCTCCCTTACTTTACCTCTCTGTCATGTGCAGATGAAGGCTCAGGGTGCTAGAGGATTAGTAAGATCTCTTTCTAAAGACAGGAGAGATTATTTACAAGAAGAACTCACCAGGGTTTAGTTTGCATTTAAGAATTGCCAGTCTTTTGTCCTGCATCATCTTGAACATTAATCCACATGTTTCAGAGCTCACCAGGCAGTACCAATGCTCTTTTCACAGCTATGAAGAGCTAGAGAAATTCTTGTTATGGTAGAAAAATTTCACGATTCATTTTTGAAACTGCATTTGTGCGTATGCAGTGTAGATTTTATAGTGTGTTGTGCTTTCAAGATCTAAATCATATATAATAAATTAAGGGACAATGGGGCTGACAGCACTAAACTTGGTGCTTATTGATATTCTAAGAAATATCTGTGAAATATCATCACGTATGTTATACAACCTTCATTTAAAAAGGTTTAAAACTAGTTAGATTCACTTTGACACTTTTCATATCATTTCTTAACCCAAGTGACGAAAACATTGTCCCCAATGAATATACTCATTAGAATTACCATTTGTTAATATCACTCATTAATTAACCCCATAATTAGATCCATTAATTTAAATGATTTAAATTTAAGTAAGTTTTATAAGGTCTGACATCAGAGGTATCTTACTTTCCTCTGAGGATGATGTACTTGCCCTGACCATGCATTTTACCATCACACATGTTCAGAAAGGGCCAAATTCCCAACCTGCTCATTTTTTTTTTATCAGAGTCATGATGAATCAGTCCTAGAATGTTTCATTTGCACAAGTAGGGCTGCCTCCAAGAGGAACCTCTGATTTATTTTGTATGAAATATATGTGAAAGGATATGAATCTGAGAGATGCTGTAGACATCTGTCCTACACTTGAGATGATTTCCAAGCCTCTCTGGCACTTTGAGTTAAGTCTATCTGGTATTAAATGCCAAGGACCTTTTGCTGCCTAAATCCACTCTGCAGGAAATAGGCCCAACCACCAGATGAGAATTAGGCCCTGGATGAGTAGCGCTATAGTTACTGTCCTGTTGATTAATTTCTGCCATTTCATGTCCATAAAAGAGACCACCCATATCATGCACACAATTAGATTTCTCACACTCTAACTGTATATTTGTATGATATTTTAAAATCTCCTAAATGCTGGGCAATGGCTATTAACAATTAATTGTCTTGCACTGGCCTTCTGATGAAATGTTAACAATGCCTATTGTAATATAGAAAAAAACATTCTATCTACTGATTTGGGCTGAATGTATGTAAATAGGTTTCTAAAAAGTCAGATGTTTGAGCAGTGGCCTACAAATCAGTAATTTTCGGATGGGAGAGTTTCTTTACATTGCCGTGGCATCTTAAAAGCTATCTTCATGTAAATTGACTGTACTAGGCCTACTGGGGATCAGAGTTCCCAAGAAAGGAAACCTTTTCTTGTATCTGGATTCAAATTTATTTCCAATGTTTCAAGCGGGAAACATGACTCTTTATTGTCTGTAAATCTAACATTATTACTTTTCCTCTTAGAAGAATATTGTATTGTTAGATGTTTGTTGAGCTGGTAACATCGTTGCAACCACTGCAATATCTTCGTTAGTAATCTGTATAATACTTTGTATACAAGTACTGGTAAGATTGTTATTAAATGTAGCTTCAGTCATTAAATTACTATAGCAAAGTAGTACTTCTTCTGTAATATTTACAATGTATTAAGCCCACAGTATATTTTATTTCAATGTAATTAAACTGTTAACTTATTCAAAGAGAAAACATCTCATCATGTCTATTGTCCAAAGTTACCTGGAATCAAATAAAAATTCTAGATTACCATGAAGAACATAAAATGCCTTTGAACTCTGCCTTATTTCACAGTCTGATGGCAAAAAATGAGGAAATTGCATCGCATTGTCTGAGTAGGTGTCATTCTATTCTGGGGGGTGGGGTGGGGCAGGACAGCAAGGGGGAGGATTGGGAAGAGAATAGCAGGCATGCTGGGGAGCGGCCGCAGGAACCCCTAGTGATGGAGTTGGCCACTCCCTCTCTGCGCGCTCGCTCGCTCACTGAGGCCGGGCGACCAAAGGTCGCCCGACGCCCGGGCTTTGCCCGGGCGGCCTCAGTGAGCGAGCGAGCGCGCAGCTGCCTGCAGGGGCGCCTGATGCGGTATTTTCTCCTTACGCATCTGTGCGGTATTTCACACCGCATACGTCAAAGCAACCATAGTACGCGCCCTGTAGCGGCGCATTAAGCGCGGCGGGTGTGGTGGTTACGCGCAGCGTGACCGCTACACTTGCCAGCGCCCTAGCGCCCGCTCCTTTCGCTTTCTTCCCTTCCTTTCTCGCCACGTTCGCCGGCTTTCCCCGTCAAGCTCTAAATCGGGGGCTCCCTTTAGGGTTCCGATTTAGTGCTTTACGGCACCTCGACCCCAAAAAACTTGATTTGGGTGATGGTTCACGTAGTGGGCCATCGCCCTGATAGACGGTTTTTCGCCCTTTGACGTTGGAGTCCACGTTCTTTAATAGTGGACTCTTGTTCCAAACTGGAACAACACTCAACCCTATCTCGGGCTATTCTTTTGATTTATAAGGGATTTTGCCGATTTCGGCCTATTGGTTAAAAAATGAGCTGATTTAACAAAAATTTAACGCGAATTTTAACAAAATATTAACGTTTACAATTTTATGGTGCACTCTCAGTACAATCTGCTCTGATGCCGCATAGTTAAGCCAGCCCCGACACCCGCCAACACCCGCTGACGCGCCCTGACGGGCTTGTCTGCTCCCGGCATCCGCTTACAGACAAGCTGTGACCGTCTCCGGGAGCTGCATGTGTCAGAGGTTTTCACCGTCATCACCGAAACGCGCGAGACGAAAGGGCCTCGTGATACGCCTATTTTTATAGGTTAATGTCATGATAATAATGGTTTCTTAGACGTCAGGTGGCACTTTTCGGGGAAATGTGCGCGGAACCCCTATTTGTTTATTTTTCTAAATACATTCAAATATGTATCCGCTCATGAGACAATAACCCTGATAAATGCTTCAATAATATTGAAAAAGGAAGAGTATGAGTATTCAACATTTCCGTGTCGCCCTTATTCCCTTTTTTGCGGCATTTTGCCTTCCTGTTTTTGCTCACCCAGAAACGCTGGTGAAAGTAAAAGATGCTGAAGATCAGTTGGGTGCACGAGTGGGTTACATCGAACTGGATCTCAACAGCGGTAAGATCCTTGAGAGTTTTCGCCCCGAAGAACGTTTTCCAATGATGAGCACTTTTAAAGTTCTGCTATGTGGCGCGGTATTATCCCGTATTGACGCCGGGCAAGAGCAACTCGGTCGCCGCATACACTATTCTCAGAATGACTTGGTTGAGTACTCACCAGTCACAGAAAAGCATCTTACGGATGGCATGACAGTAAGAGAATTATGCAGTGCTGCCATAACCATGAGTGATAACACTGCGGCCAACTTACTTCTGACAACGATCGGAGGACCGAAGGAGCTAACCGCTTTTTTGCACAACATGGGGGATCATGTAACTCGCCTTGATCGTTGGGAACCGGAGCTGAATGAAGCCATACCAAACGACGAGCGTGACACCACGATGCCTGTAGCAATGGCAACAACGTTGCGCAAACTATTAACTGGCGAACTACTTACTCTAGCTTCCCGGCAACAATTAATAGACTGGATGGAGGCGGATAAAGTTGCAGGACCACTTCTGCGCTCGGCCCTTCCGGCTGGCTGGTTTATTGCTGATAAATCTGGAGCCGGTGAGCGTGGAAGCCGCGGTATCATTGCAGCACTGGGGCCAGATGGTAAGCCCTCCCGTATCGTAGTTATCTACACGACGGGGAGTCAGGCAACTATGGATGAACGAAATAGACAGATCGCTGAGATAGGTGCCTCACTGATTAAGCATTGGTAACTGTCAGACCAAGTTTACTCATATATACTTTAGATTGATTTAAAACTTCATTTTTAATTTAAAAGGATCTAGGTGAAGATCCTTTTTGATAATCTCATGACCAAAATCCCTTAACGTGAGTTTTCGTTCCACTGAGCGTCAGACCCCGTAGAAAAGATCAAAGGATCTTCTTGAGATCCTTTTTTTCTGCGCGTAATCTGCTGCTTGCAAACAAAAAAACCACCGCTACCAGCGGTGGTTTGTTTGCCGGATCAAGAGCTACCAACTCTTTTTCCGAAGGTAACTGGCTTCAGCAGAGCGCAGATACCAAATACTGTCCTTCTAGTGTAGCCGTAGTTAGGCCACCACTTCAAGAACTCTGTAGCACCGCCTACATACCTCGCTCTGCTAATCCTGTTACCAGTGGCTGCTGCCAGTGGCGATAAGTCGTGTCTTACCGGGTTGGACTCAAGACGATAGTTACCGGATAAGGCGCAGCGGTCGGGCTGAACGGGGGGTTCGTGCACACAGCCCAGCTTGGAGCGAACGACCTACACCGAACTGAGATACCTACAGCGTGAGCTATGAGAAAGCGCCACGCTTCCCGAAGGGAGAAAGGCGGACAGGTATCCGGTAAGCGGCAGGGTCGGAACAGGAGAGCGCACGAGGGAGCTTCCAGGGGGAAACGCCTGGTATCTTTATAGTCCTGTCGGGTTTCGCCACCTCTGACTTGAGCGTCGATTTTTGTGATGCTCGTCAGGGGGGCGGAGCCTATGGAAAAACGCCAGCAACGCGGCCTTTTTACGGTTCCTGGCCTTTTGCTGGCCTTTTGCTCACATGT

**Amino acid Sequence of the chimeric proteins GLuc–FLAG–CB1 (667 AA)**

MGVKVLFALICIAVAEAKPTENNEDFNIVAVASNFATTDLDADRGKLPGKKLPLEVLKEMEANARKAGCTRGCLICLSHIKCTPKMKKFIPGRCHTYEGDKESAQGGIGEAIVDIPEIPGFKDLEPMEQFIAQVDLCVDCTTGCLKGLANVQCSDLLKKWLPQRCATFASKIQGQVDKIKGAGGDGLEDYKDDDDKKSILDGLADTTFRTITTDLLYVGSNDIQYEDIKGDMASKLGYFPQKFPLTSFRGSPFQEKMTAGDNPQLVPADQVNITEFYNKSLSSFKENEENIQCGENFMDIECFMVLNPSQQLAIAVLSLTLGTFTVLENLLVLCVILHSRSLRCRPSYHFIGSLAVADLLGSVIFVYSFIDFHVFHRKDSRNVFLFKLGGVTASFTASVGSLFLTAIDRYISIHRPLAYKRIVTRPKAVVAFCLMWTIAIVIAVLPLLGWNCEKLQSVCSDIFPHIDETYLMFWIGVTSVLLLFIVYAYMYILWKAHSHAVRMIQRGTQKSIIIHTSEDGKVQVTRPDQARMDIRLAKTLVLILVVLIICWGPLLAIMVYDVFGKMNKLIKTVFAFCSMLCLLNSTVNPIIYALRSKDLRHAFRSMFPSCEGTAQPLDNSMGDSDCLHKHANNAASVHRAAESCIKSTVKIAKVTMSVSTDTSAEAL

**Supplementary Table. 1.** One-way ANOVA analysis with Tukey post hoc test conducted for permeabilized (P) and non-permeabilized (NP) wild type (WT), GLuc-FLAG-CB1^F237L^ (F237L), GLuc- FLAG-CB1^T210I^ (T210I) and GLuc- FLAG-CB1^T210A^ (T210A) cells. The analysis was performed using GraphPad Prism 8.0. (*, p < 0.05; **, p < 0.01; ***, p < 0.001 was considered statistically significant).

**References**

1. Verspurten J, Gevaert K, Declercq W, Vandenabeele P. SitePredicting the cleavage of proteinase substrates. Trends Biochem Sci. 2009 Jul; 34(7):319-23. PudMed
